# Indirect 3D printing technology for the fabrication of customised β-TCP/chitosan scaffold with the shape of rabbit radial head—an in vitro study

**DOI:** 10.1186/s13018-019-1136-7

**Published:** 2019-04-11

**Authors:** Ji-Qi Wang, Bing-Jie Jiang, Wei-Jun Guo, You-Ming Zhao

**Affiliations:** 1 Department of Orthopaedics, The Second Affiliated Hospital and Yuying Children’s Hospital of Wenzhou Medical University, 109# Xue Yuan Xi Road, Wenzhou, 325000 Zhejiang China; 2 Key Laboratory of Orthopedics of Zhejiang Province, Wenzhou, 325000 Zhejiang China; 3 The Second School of Medicine, Wenzhou Medical University, Wenzhou, 325000 Zhejiang China

**Keywords:** Three-dimensional printing, Scaffold, Tissue engineering, β-TCP, Chitosan, Customised

## Abstract

**Background:**

With the development of indirect three-dimensional (3D) printing technology, it is possible to customise individual scaffolds to be used in bone transplantation and regeneration. In addition, materials previously limited to the 3D printing (3DP) process due to their own characteristics can also be used well in indirect 3DP. In this study, customised β-TCP/chitosan scaffolds with the shape of rabbit radial head were produced by indirect 3D printing technology.

**Methods:**

Swelling ability, porosity, mechanical characterisation, and degradation rate analysis were performed, and in vitro studies were also implemented to evaluate the proliferation and osteogenic differentiation of bone marrow mesenchymal stem cells (MSCs) on the scaffolds. CCK8 cell proliferation assay kit and alkaline phosphatase (ALP) staining solution were used to study cell proliferation and early ALP content at the scaffold surface. Moreover, the osteogenic differentiation of MSCs on scaffolds was also evaluated through the scanning electron microscopy analysis.

**Results:**

β-TCP/chitosan scaffold has good performance and degradation rate, and in vitro cell experiments also confirm that the scaffold has adequate cytocompatibility and bioactivity.

**Conclusion:**

This study provides a promising new strategy for the design of customised scaffolds for the repair of complex damaged tissues.

## Background

Radial head fracture is one of the most common elbow fractures in adults and accounts approximately one third of all cases [1]. There are many treatment methods for this type of fracture at present, such as Kirschner wires, hollow screw, plate, and radial head arthroplasty (RHA) [2]. As far as we know, the indication for RHA is the unreconstructable radial head [3–5]. However, previous studies have shown that the functional recovery of some patients after RHA is unsatisfactory. Moreover, there are many complications, including prosthetic loosening, wear and tear between the prosthesis and the humerus, and chronic pain caused by the prosthesis, all resulting in increased mechanical stress on the surrounding tissue, inflammation, and even requiring reoperation [6–8].

Clinically, RHA has become increasingly mature. However, there is widespread controversy in the design and application of specific prostheses. Gupta et al. [9] suggested that the present radial head prosthesis does not conform to normal anatomy and that its design is not based on the geometric parameters of the radial head and proximal medulla of the radius. Beredjiklian et al. [10] used MRI to measure the anatomical parameters of the radial head and found that the design parameters of the stem of the radial head prosthesis were larger than the intramedullary diameter of the proximal radius. Many complications might be owed to the failure of the present prosthesis to achieve the normal anatomical shape and size of the radial head and to the poor anatomical matching of the prosthesis to the elbow joint. The shape of the rabbit radial head is complex, and it is difficult to replicate with ready-made prostheses [11]. Rabbit radial head is similar to human radial head, where it is elliptical and the joint disc is eccentric relative to the neck. Previous studies have shown that the contact surface shape of elbow prosthesis is significantly correlated with the corresponding pressure during elbow movement [12, 13]. Therefore, it is necessary to design an individualised prosthesis for patients.

In recent years, there has been a rapid development of clinical digital assistant technology, providing accurate measurement of the anatomical parameters of the radial head and making it possible for the development of an individual prosthesis based on the anatomical structure of the patient [14, 15]. Previous studies have made individual joint prostheses through digital assistant technology for application in replacement therapy for hip and knee joints [16, 17]. However, even if a radial head prosthesis is designed to fit the anatomy of the elbow joint, it may not be able to solve the problems of prosthetic loosening, wear and tear between the prosthesis and the humerus, and longevity of the prosthesis. At this point, a bioactive prosthetic replacement material with high bioactivity and controllable degradability is needed to replace the current materials used in clinical practice to solve the above problems.

Normally, the skeletal system consists mainly of cortical bone and trabecular bone. Cortical bone accounts for about 80% of the total, with compact structure and low surface area forming a capsule around the bone marrow cavity. In contrast, trabecular bone accounts for about 20% of the total, consists of a network of interconnected trabeculae, and is separated by a space filled with bone marrow [18]. Normal skeletal system maintains dynamic balance and stability under the action of osteocytes, osteoblasts, and osteoclasts. However, pathological skeleton is prone to osteoporosis and pathological fracture due to some factors such as when the speed of bone absorption is faster than the speed of bone formation, which leads to the decrease of bone trabecula and bone density [19, 20]. As far as we know, biological scaffolds could guide cell growth and differentiation to the conceived regenerated tissue. Thus, healthy cells can gradually replace natural extracellular matrix scaffolds during biosynthesis [21, 22]. Researchers have been looking for an ideal bone substitute material which can complete mechanical support for bone tissue within a specific time of injury healing. Moreover, it should be non-toxic with no residue to the human body, thereby avoiding the effect of foreign bodies on human tissues [23–27].

Calcium phosphate ceramics are widely used in clinical practice because of their good stability and non-toxic degradation process [28–30]. Among calcium phosphate ceramics, β-tricalcium phosphate (β-TCP) could be dissolved under acidic conditions caused by released cells, such as osteoclasts or macrophages Moreover, its degradation rate is ten times higher than hydroxyapatite [31]. However, there are still some deficiencies in the porous β-TCP scaffolds: First, mechanical properties are insufficient, brittleness is large, and bending resistance is poor; thus, they cannot be used as a repair for the weight-bearing bone defect. Second, there is a lack of inducing activity, and they can only rely on bone conduction to make bone tissue growth. Moreover, osteogenesis is insufficient, and the depth of osteogenesis is limited. Lastly, the degradation rate is difficult to control and does not match the growth rate of new bone [32–34].

Chitosan (CS), as a biopolymer, has attracted much attention due to its biocompatibility and biodegradability, and it has been appreciated in bone tissue engineering [35]. In addition, chitosan used in the production of the scaffolds can interact with negatively charged substances on the surface of bacteria and increase cell wall permeability, thereby playing a bactericidal role [36]. Previous studies have demonstrated that chitosan/β-TCP scaffolds have good mechanical and biological properties and can be used as a potential bone repair scaffold [37–39].

Solid freeform fabrication (SFF) is a general term that includes the manufacture of various structures through computer-aided design (CAD) [40]. In addition, other terms are usually used to describe the same principle, such as additive manufacturing (AM), rapid prototyping (RP), and 3D printing (3DP) [40]. SFF, based on medical imaging techniques, can reproduce tissue defects that mimic a particular patient. However, this technology is limited by the range of materials available for processing [41–43]. To overcome this limitation, indirect rapid prototyping (iRP) was proposed, which produces a mould or template for the final scaffold structure using SFF technology [44–46]. The final scaffold parameter requirements are secondary because the mould is a straightforward process of well-established materials [21, 47]. Consequently, due to the thermal mechanical properties not matching the usual temperature and pressure requirements of direct rapid prototyping, some materials are identified as “unprintable”, and well-defined three-dimensional (3D) scaffolds can be generated through this approach [48–50]. In addition, iRP allows a direct combination of different materials in a scaffold, including bioactive compounds [42, 50].

In our study, we customised theβ-TCP/chitosan scaffold with the shape of New Zealand rabbit radial head through iRP technology, using a negative mould. The final scaffold was produced from a negative replica of a desired radial head shape, then the structure, performance, degradation, and ability to induce new bone formation of the scaffold were tested. The purpose of our study is to provide a promising new strategy for the design of customised scaffolds for the repair of complex damaged tissues and a theoretical basis for the feasibility of making a customised, biodegradable, and bioceramic radial head prosthesis that conforms to human anatomy, which can be used in the human body.

## Methods

### Materials

Chitosan (degree of deacetylation > 85%), β-TCP powder (≥ 98% β-phase basis), paraffin (melting point between 53 and 57 °C), N-hexane, Tris buffer (pH = 7.4), ethanol (EtOH), phosphate-buffered saline (PBS) solution, streptomycin, gentamicin, dexamethasone, ascorbic acid, β-polyglycerolphosphate disodium, alkaline phosphatase (ALP) staining solution, and glutaraldehyde were purchased from Sigma-Aldrich (St. Louis, MO, USA). Trypsin/EDTA solution, Dulbecco’s modified Eagle’s medium (DMEM; low-glucose and high-glucose), and foetal bovine serum (FBS) were obtained from Gibco (Grand Island, NY, USA). CCK8 cell proliferation assay kit was purchased from Dojindo (Kumamoto, Japan).

### Design and fabrication

**Fig. 1:**
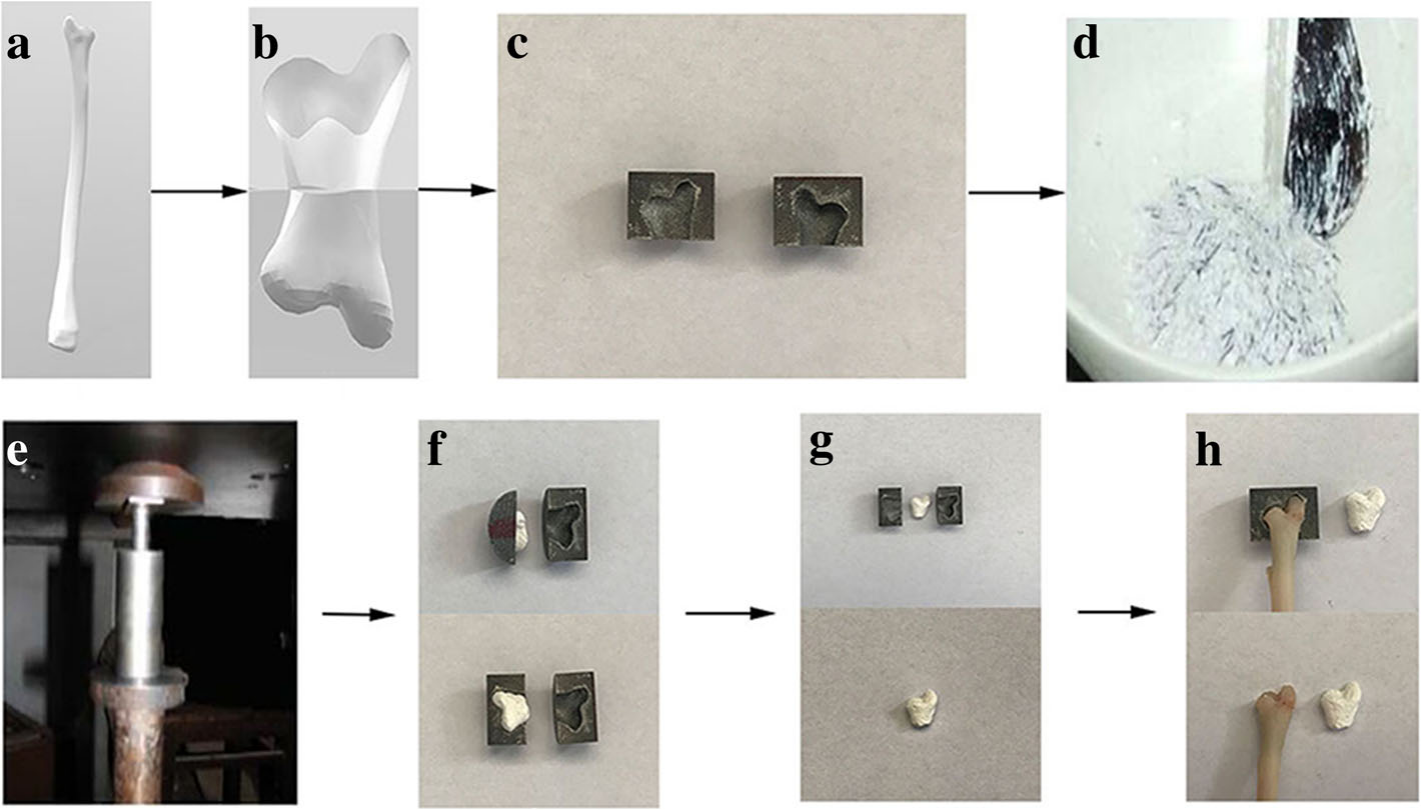
Scaffold fabrication process by indirect 3DP. **a** 3D reconstruction of rabbit radial head from CT images. **b** Creating a negative replica mould using CAD modelling, divided into two parts. **c** 3D printed the negative replica mould. **d** Preparation of polymer solutions. **e** The polymer solution was poured into a negative replica mould with radial head shape and compacted. **f**, **g** Demoulding, removal of paraffin microspheres, and freeze-drying of the scaffold. **h** Comparison between rabbit radial head and scaffold

The procedure for mould design and scaffold fabrication is shown in Fig. 1. Briefly, the anatomy of the radial head was separated from CT scans using commercially available software (Mimics, Materialise, Ann Arbor, MI, USA), and a negative replica mould of a desired radial head shape was modelled using computer-aided design (CAD). The image data defined were rendered by 3D and converted to STL file for 3D printing (3DP). The object was cut into two-dimensional (2D) layers, and each separate slice layer was constructed on a commercially available 3DP machine as previously described [46, 51]. Generally, the negative replica 3D mould is made of silicon [52].

Chitosan was dissolved in acetic acid (0.1 M) to form a 2% (*w*/*v*) solution. The solution was then mixed through an overhead mixer (IKA T25 Ultra Turrax) for 2 min to obtain a transparent gel. β-TCP powder (0.4% *w*/*v*) was dispersed in ethanol solution for 1 h to obtain a paste mixture. The paste was then added into chitosan solution and homogenised by magnetic stirrer. Paraffin microspheres (approximately 300 μm) were prepared by dispersion method and used as a porogen in the production of the scaffolds [53, 54]. Then, paraffin microspheres were added to the mixture of β-TCP and CS solution to form a suspension and stirred overnight [55]. Afterwards, the polymer solution was poured into a negative replica mould with radial head shape and compacted. Next, it was placed at − 20 °C for 24 h, then freeze-dried for another 24 h. Finally, the scaffolds were taken from the mould, and the porogen was removed using Soxhlet extractor for 24 h, with N-hexane as the refluxing solvent [56]. All the scaffolds were vacuum-dried at 40 °C for 6 h and stored at − 20 °C for further studies.

### Swelling ability

The swelling ability of the scaffold was measured in Tris buffer (pH = 7.4) at 37 °C for up to 24 h [57]. At each time point, the swollen sample was removed from the solution, the excess of Tris buffer was removed with filter paper, and the weight of the sample was measured. Five samples were used for each measurement, and the average value was obtained and used for analysis. The swelling ratio was determined using the following formula:

$$ \mathrm{Swelling}\ \mathrm{ratio}\ \left(\%\right)=\left({W}_{\mathrm{s}}-{W}_{\mathrm{d}}\right)/{W}_{\mathrm{d}}\times 100 $$

where *W*_s_ is the swollen weight of scaffold, and *W*_d_ is the initial dry weight of scaffold.

### Porosity evaluation

A liquid displacement method was used to determine the porosity (*P*) of the scaffold [58]. Ethanol (EtOH) was selected due to its inability to swell or shrink the matrix when permeating through the scaffold. The scaffold was immersed in EtOH for 48 h, and the porosity was determined using the following formula:

$$ P\left(\%\right)=\left({W}_2-{W}_1\right)/\left({d}_{\mathrm{ethanol}}\times {V}_{\mathrm{scaffold}}\right)\times 100. $$

where *W*_2_ and *W*_1_ represent the wet and dry weight of the scaffold, respectively, while *d*_ethanol_ and *V*_scaffold_ represent the density of the ethanol and the volume of the scaffold, respectively. Five samples were used for each measurement, and the average value was obtained and used for analysis.

### Mechanical properties

Uniaxial compression assays with 1000 N of loaded cell and 1 mm min^−1^ crosshead speed conditions were performed to test the compressive strength of the scaffold [59]. Five samples were used, and compressive strength (*S*) was determined using the following formula:

$$ S={F}_{\mathrm{max}}/A $$

where *F*_max_ and *A* denote the maximum applied load and the initial contact area of the scaffold, respectively. Average values and standard deviation (SD) were determined.

### Determination of degradation rate

The degradation of scaffold was performed through an adaptation of the method previously used by Kim and collaborators [60], which measured a simulated body fluid (SBF) containing lysozyme. At predetermined intervals, the scaffold was lyophilised and the final dry weight (*W*_d_) was recorded. Five samples were used for each measurement, and the average value was obtained and used for analysis. The degradation of scaffold was determined using the following formula:

$$ \mathrm{Degradation}\ \mathrm{rate}\ \left(\%\right)=\left({W}_{\mathrm{I}}-{W}_{\mathrm{d}}\right)/{W}_{\mathrm{I}}\times 100. $$

where *W*_I_ and *W*_d_ represent the initial and final dry weight of the scaffold, respectively.

### Cell culture and seeding

All animal procedures and protocols were reviewed and approved by the Animal Research Committee of the Wenzhou Medical University. According to the method previously reported by Hokugo et al. [61], bone marrow mesenchymal stem cells (MSCs) were isolated from the iliac bone marrow aspirates of New Zealand white rabbits. Briefly, the density gradient centrifugation method was performed to isolate the MSCs. Then, MSCs were cultured at 37 °C in a humidified atmosphere containing 5% CO_2_. DMEM low-glucose supplemented with foetal bovine serum (FBS, 10% *v*/*v*), streptomycin (100 μg ml^−1^), and gentamicin (100 μg ml^−1^) were used as growth medium, which was changed every 3 days. When reaching about 80–90% confluence, the cells were trypsinised and passaged for further expansion, and the third passage was used in the following experiments. Prior to cell seeding, ultraviolet bactericidal was performed for 30 min, followed by EtOH immersion for 60 min for the scaffolds and a wash step with PBS three times [62]. An optical microscope was used to monitor the growth of MSCs.

### Cell viability

Each scaffold was seeded with 50 μl of cell suspension at a concentration of 1 × 10^6^ cells ml^−1^. After incubation and adhesion for 2 h at 37 °C, the scaffolds were placed in a 24-well plate and cultured with 1 ml growth media (DMEM low-glucose/10% FBS). At each time point (24, 48, and 72 h), cell activity in the scaffold was measured by CCK8 cell proliferation assay kit, following the manufacturer’s instruction. After washing with PBS, the scaffolds (*n* = 5) were incubated in 180 μl of fresh medium with 20 μl of CCK8 assay solution in 24-well plates for 1 h. A Multiskan Spectrum (Thermo Fisher Scientific, USA) at a wavelength of 450 nm was used to measure the optical density of each well.

### Alkaline phosphatase staining and scanning electron microscopy analysis for osteogenic differentiation

Each scaffold was seeded with 50 μl of cell suspension at a concentration of 1 × 10^6^ cells ml^−1^. After incubation and adhesion for 2 h at 37 °C, the scaffolds were placed in a 24-well plate and cultured with 1 ml growth media (DMEM low-glucose/10% FBS) for 24 h then cultured in osteogenic medium, which was changed every 2 days. The osteogenic medium was DMEM high-glucose supplemented with 5% FBS, 0.1 μM dexamethasone, 50 μM ascorbic acid, 10 mM β-polyglycerolphosphate disodium, 100 μg ml^−1^ streptomycin, and 100 μg ml^−1^ gentamicin. After incubation for 7 days, the osteogenic differentiation scaffold was washed with serum-free DMEM high-glucose for 8 h to remove serum components, then with PBS three times. Next, the scaffolds were immersed in stationary liquid for 2 h. Afterwards, the immobilised scaffolds were immersed in ALP staining kit for 2 h according to the manufacturer’s instruction for.

For scanning electron microscopy (SEM) analysis, at each time point (incubation for 14 and 28 days), the post-seed scaffolds were washed with PBS and fixed overnight with 2.5% (*v*/*v*) glutaraldehyde. Then, the scaffolds were washed three times in PBS and dehydrated in grading EtOH solutions (50, 60, 70, 80, 90, and 99.9%). Liquid nitrogen was used to freeze the scaffolds, which were freeze-dried for 3 h then mounted onto aluminium stubs with double adhesive tape and sputter-coated with gold using a Quorum Q150R ES sputter coater. SEM images were obtained with an acceleration voltage of 20 kV at variable magnifications using a Hitachi S-3400 N Scanning Electron Microscope [63].

## Results

**Fig. 2:**
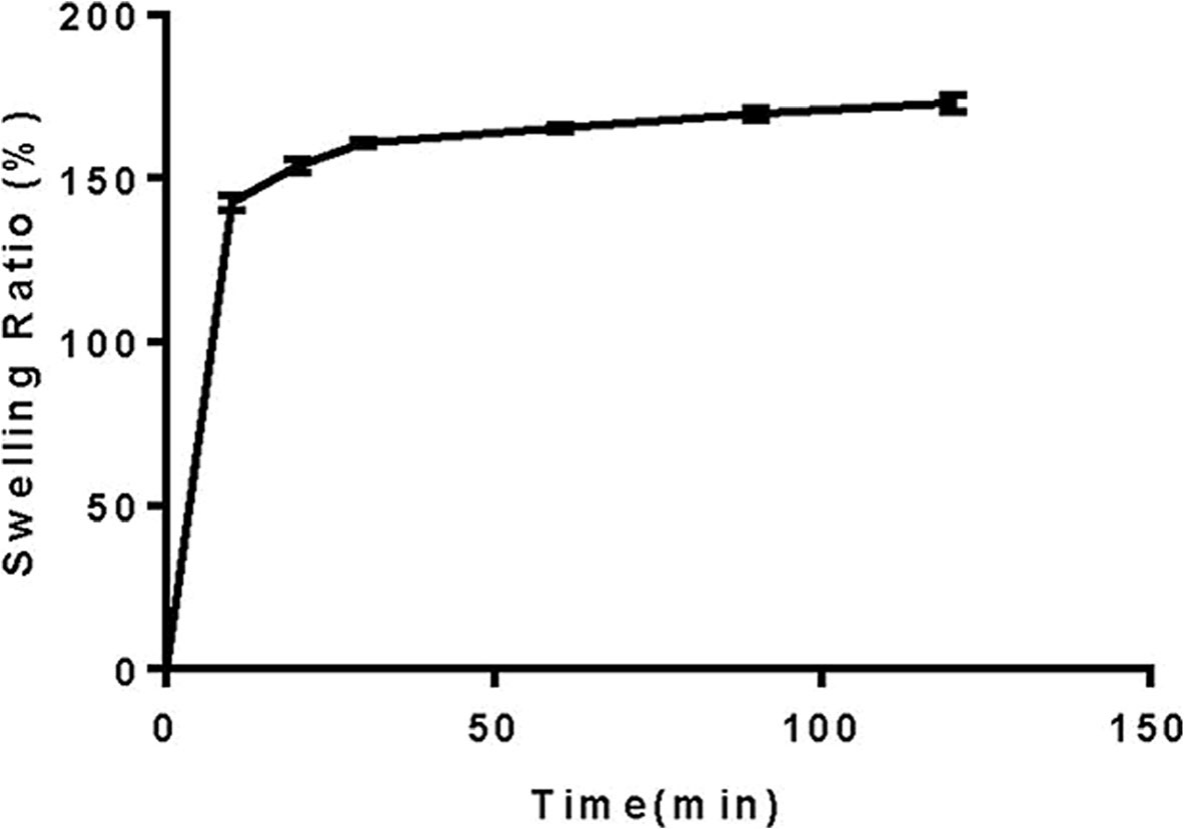
Swelling profile of the scaffold produced

Figure 1 shows a fabricated mould with an external shape matching the radial head of New Zealand rabbit through 3D printing technology. In this study, the swelling ability of the scaffold in PBS is shown in Fig. 2. The rate of increase is highest after 30 min, and then the change tends to stabilise. We used the liquid displacement method to evaluate the porosity of scaffolds. The porosity of all scaffolds was higher than 75%, and the mean porosity was 78.98 ± 2.42%. Then, we used a uniaxial compression test to evaluate the compressive strength of the scaffolds. The mean compressive strength was 1.73 ± 0.09 MPa. Moreover, we found that the mean mass loss of scaffold in the first month was 15.70 ± 1.24%.

**Fig. 3:**
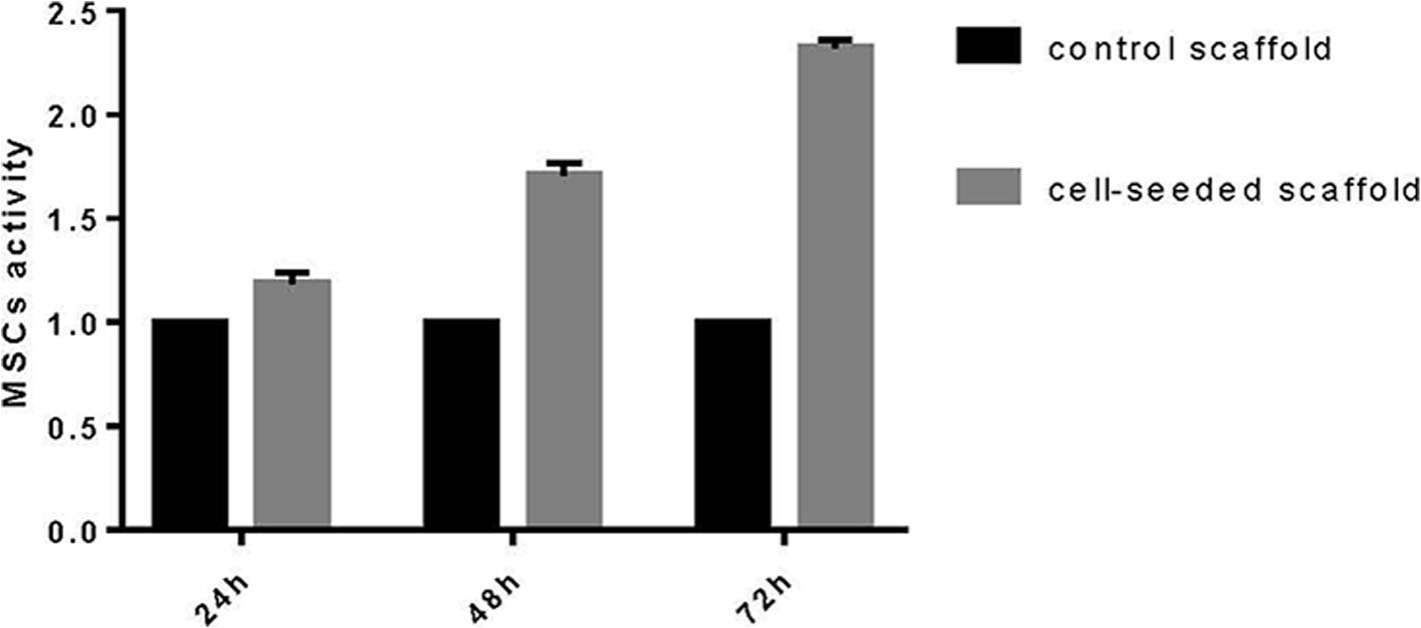
Evaluation of cell viability in the presence of the scaffolds. Cell viability was evaluated after 24, 48, and 72 h using a CCK8 assay. Scaffolds without cell seeding were used as a control group

**Fig. 4:**
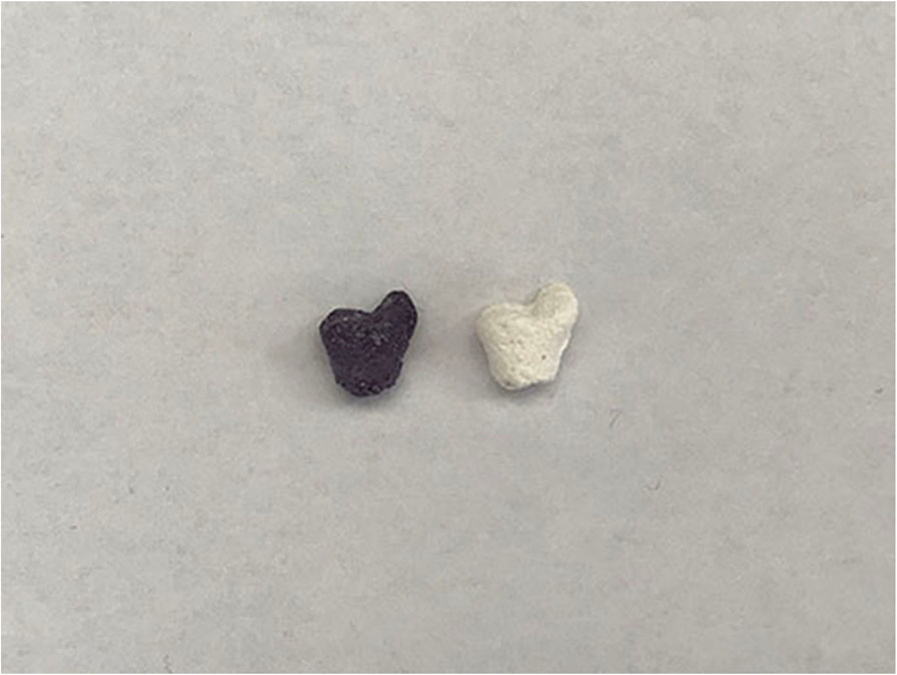
Alkaline phosphatase (ALP) staining. The existence of ALP was tested in early osteogenic differentiation of MSCs in the presence of the scaffolds after 7 days using an ALP staining solution. Left: alkaline phosphatase stained after 7 days of incubation with MSCs. Right: control after 7 days in the same media without MSCs and stained

**Fig. 5:**
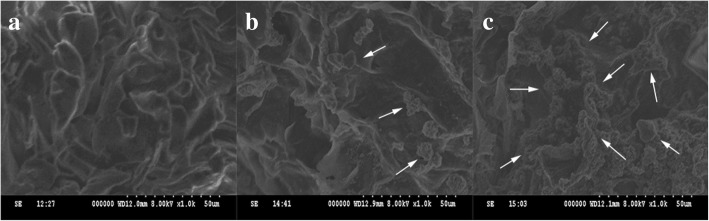
The surface topographical features of cells on scaffold imaged by scanning electron microscopy (SEM). **a** SEM image of β-TCP/chitosan scaffold. **b** After 14 days of incubation in osteogenic medium, the cells contacted to each other and spread on the surface of the scaffold. Moreover, spherical and polygonal adhesion on the surface and around the pores of the scaffold were observed (arrow). **c** On day 28, large patches and crumby structure of adhesions were seen on the surface and around the pores of the scaffolds, which was increased significantly compared to 14 days prior (arrow)

Cell viability on scaffold was characterised using a CCK8 cell proliferation assay kit (Fig. 3). ALP staining suggests that MSCs could induce osteogenic differentiation on the scaffold within osteogenic differentiation medium at an early stage (Fig. 4). Finally, the surface topographical features of cells on scaffold were imaged by SEM (Fig. 5).

## Discussion

As far as we know, many similar studies have used different methods to prepare polymer/bioceramic composite materials [64, 65]. For example, Heydary et al. [66] prepared polyvinyl alcohol (PVA)/Iranian gum tragacanth (IGT) nanocomposite fibre by electrospinning (ELS) technique. Furthermore, Khandan et al. [67] synthesised magnetite nanoparticles by co-precipitation method and a novel bredigitemagnetite nanocomposite by mechanical grinding and subsequent sintering process combined with bredigite powders. In our study, β-TCP/chitosan scaffold is a type of composite bioceramics that acts as osteoconduction and structural support. Complex three-dimensional anatomical scaffolds were fabricated by indirect 3DP technology combined with imaging technology, and CT data were used to design the anatomical radial head. In tissue engineering scaffolds, porogen was added to enhance the transport of oxygen and nutrients to maintain cell viability and promote vascular endogenous growth in the graft [68, 69]. Murphy et al. [70] studied the effect of 85–325-μm pore size on cell activity and found that the number of cells at the maximum pore size were always the highest, prompting them to define 325 μm as the optimal pore size for cell proliferation. Moreover, it has been reported that pore size greater than 300 μm was essential for bone ingrowth and scaffold vascularisation [71]. Scaffolds were produced by pouring the polymer solution into the printed mould with radial head shape and compacted.

### In vitro performance testing

Fluid absorption capacity is necessary for scaffolds in tissue engineering [72], and satisfactory swelling ability not only promotes cell attachment but also cell internalisation, which is the basis for improving tissue regeneration process. In addition, high swelling ability can also promote the diffusion of nutrients and wastes along the structure [73]. However, excessive swelling ability leads to loss of mechanical integrity and compressive stress of surrounding tissues [74]. In our study, the rate of increase is highest after 30 min, then the change tends to stabilise, which means scaffold has a satisfactory fluid absorption capacity. The porous structure of scaffolds is of great significance for cell proliferation, differentiation, and function [75]. The porosity of all scaffolds was greater than 75%, and the mean porosity was 78.98 ± 2.42%. In fact, interconnected pores promote the formation of vascular networks in scaffolds and provide channels for free diffusion of ions, nutrients, and cells. The diffusion of calcium ion and phosphate anion in the scaffold can produce a layered material similar to hydroxyapatite (HA), which stimulates the activity of osteoblasts [59]. Additionally, the HA-like layer can also increase bone conduction and osseointegration, further improving bone mineralisation [76]. As far as we know, the characterisation of compressive strength is very important for the design and production of bone tissue engineering scaffolds [77, 78]. The mean compressive strength was 1.73 ± 0.09 MPa. Our results show that the mechanical strength of the produced scaffold is lower than that of trabecular bone (2–20 MPa) [79]. Actually, the scaffold similar to this kind of production only needs to play a template in the first stage of bone regeneration. When the scaffold begins to be biodegraded, it will be replaced by the new bone matrix, which is compatible with the formation of the new bone tissue [77, 80]. These characteristics enable it to provide temporary support in the mineralisation stage, accelerate the regeneration process, and improve the mechanical capability of the target position [81]. Our preliminary data are consistent with the results of Siddiqui et al. [82], and we found the mean mass loss of scaffold in the first month was 15.70 ± 1.24%, indicating that the scaffold has good degradation ability. When the degradation rate of the scaffold matches the osteogenesis rate of the new bone, the scaffold can provide adequate temporary support.

### Cell viability and osteogenic differentiation

Cell viability on scaffold was characterised through a CCK8 cell proliferation assay kit. The results demonstrate that with the increase of culture time, MSCs can proliferate on scaffold satisfactorily. Then, we tested the ALP activity, an early indicator of MSCs induced to osteoblasts, through an ALP staining solution. The results suggest that MSCs could induce osteogenic differentiation on the scaffold within osteogenic differentiation medium at an early stage. Finally, the surface topographical features of cells on scaffold were imaged by scanning electron microscopy (SEM). After 14 days of incubation in osteogenic medium, the cells contacted to each other and spread on the surface of the scaffold. Moreover, spherical and polygonal adhesion on the surface and around the pores of the scaffold were observed. On day 28, large patches and crumby structure of adhesions were noted on the surface and around the pores of the scaffolds, which was increased significantly compared to 14 days prior. The images showed that the proliferation and osteogenic differentiation of the cells increased with culture time.

## Conclusions

In this study, we demonstrated an indirect 3DP technology to create a customised scaffold with anatomic radial head shape. Briefly, β-TCP and chitosan polymer solution were mixed with paraffin microspheres porogens and poured into a negative replica mould, which was produced by indirect 3DP technology. Subsequently, a leaching technique was used to fabricate the final porous scaffold. We found that the scaffold has good performance and degradation rate. Moreover, in vitro cell experiments also confirm that the scaffold has adequate cytocompatibility and bioactivity. Further studies are required to determine whether this scaffold supports tissue regeneration in vivo. This study provides a promising new strategy for the design of customised scaffolds for the repair of complex damaged tissues.

## Abbreviations

3DP: 3D printing
ALP: Alkaline phosphatase
AM: Additive manufacturing
CAD: Computer-aided design
CS: Chitosan
DMEM: Dulbecco’s modified Eagle’s medium
FBS: Foetal bovine serum
HA: Hydroxyapatite
iRP: Indirect rapid prototyping
MSCs: Mesenchymal stem cells
PBS: Phosphate-buffered saline solution
RHA: Radial head arthroplasty
RP: Rapid prototyping
SBF: Simulated body fluid
SEM: Scanning electron microscopy
SFF: Solid freeform fabrication
β-TCP: β-tricalcium phosphate
